# A six-inhibitor culture medium for improving naïve-type pluripotency of porcine pluripotent stem cells

**DOI:** 10.1038/s41420-019-0184-4

**Published:** 2019-06-17

**Authors:** Ye Yuan, Jinkyu Park, Yuchen Tian, Jungmin Choi, Rolando Pasquariello, Andrei P. Alexenko, Aihua Dai, Susanta K. Behura, R. Michael Roberts, Toshihiko Ezashi

**Affiliations:** 1 Bond Life Sciences Center, University of Missouri, Columbia, MO 65211 USA; 2 Division of Animal Sciences, University of Missouri, Columbia, MO 65211 USA; 3 Colorado Center for Reproductive Medicine, Lone Tree, CO 80124 USA; 4 Department of Internal Medicine, Yale School of Medicine, New Haven, CT 06510 USA; 5 Laboratory of Human Genetics and Genomics, The Rockefeller University, New York, NY 10065 USA; 6 Department of Agricultural and Environmental Sciences—Production, Landscape, Agroenergy, University of Milan, Milano, 20133 Italy

## Abstract

Understanding essential signaling network requirements and making appropriate adjustments in culture conditions are crucial if porcine pluripotent stem cells (PSC) are to achieve their full potential. Here, we first used two protein factors (LIF and FGF2) and kinase inhibitor combinations in attempts to convert primed type lentiviral-reprogrammed porcine induced PSC (Lv-piPSC) into naïve-like state and developed a medium called FL6i. In addition to FGF2 and LIF, this medium contained inhibitors of MAPK14, MAPK8, TGFB1, MAP2K1, GSK3A and BMP. Crucially, the usual TGFB1 and BMP4 protein components of many stem cell media were replaced in FL6i with inhibitors of TGFB1 and BMP. With this medium, Lv-piPSC were readily transformed from their original primed state into cells that formed colonies with typical features of naïve-state stem cells. The FL6i medium also assisted generation of naïve-type piPSC lines from porcine embryonic fibroblasts with non-integrating episomal plasmids (Epi-piPSC). These lines, despite retaining variable amounts of vector DNA, expressed higher endogenous *pPOU5F1* and *pSOX2* than Lv-piPSC. They have been cultured without obvious morphological change for >45 passages and retained pluripotent phenotypes in terms of upregulation of genes associated with pluripotency, low expression of genes linked to emergence of somatic cell lineages, and ability to generate well differentiated teratomas in immune-compromised mice. FL6i conditions, therefore, appear to support elevated pluripotent phenotypes. However, FL6i was less able to support the generation of embryonic stem cells from porcine blastocysts. Although colonies with dome-shaped morphologies were evident and the cells had some gene expression features linked to pluripotency, the phenotypes were ultimately not stable. Pathway analysis derived from RNAseq data performed on the various cell lines generated in this study suggest the benefits of employing the FL6i medium on porcine cells reside in its ability to minimize TGFB1 and BMP signaling, which would otherwise de-stabilize the stem cell state.

## Introduction

Pigs probably constitute the premier non-primate model for biomedical testing because of their physiological similarities to humans^1^. Accordingly, porcine pluripotent stem cells (PSC) potentially provide powerful tools for evaluating the safety and efficacy of stem cell based therapies, as well as having utility in the production of transgenic pigs and xenografts^1,2^. However, the derivation of authentic porcine embryonic stem cell (pESC)^3–8^ and transgene free porcine iPSC (piPSC)^9–12^ that can readily differentiate into all cell lineages still remains problematic^13^, although several promising advances have recently been reported^10,14–16^.

Most, if not all, piPSC lines depend on ectopic genes expressions to maintain self-renewal because of a failure to fully activate the endogenous genes required to provide a pluripotent phenotype^7,17–19^. The persistent expression of these reprogramming genes may complicate cell differentiation protocols, and there is concern that they may produce tumors in the treated animal. Various non-viral reprogramming methods have been tested to obtain transgene-free piPSC, but outcomes fail to up-regulate endogenous genes and whether or not the persistence of ectopic gene expression is required for pluripotency have been ambiguous^18,20–22^. There have also been consistent failures in generating pESC from the inner cell mass (ICM) and epiblast of porcine embryos, suggesting that special culture conditions are necessary to maintain stable endogenous pluripotency networks in pig cells. It has long been known that supplementation with certain cytokines and small molecule inhibitors and other fine tuning of the culture conditions can allow derivation of PSC from certain “non-permissive” mouse strains and rats^23–25^ and{Buehr, 2008 #141} permit generation of human PSC in various states of pluripotency^26–32^.

Here we report the development of culture conditions, based on the naïve human stem cell medium (NHSM)^27^, that permits conversion of primed/epiblast-types of piPSC into cultures with naïve-type properties. Two other goals were to employ similar culture conditions to produce piPSC from somatic cells by using non-integrating episomal plasmids and to generate genuine pESC from outgrowths of porcine blastocysts.

## Materials and methods

### Routine maintenance and culture conditions of PSC

For routine maintenance, piPSC and pESC like cells (pESCLC) were cultured on irradiated mouse embryonic fibroblasts (iMEF) in 10 cm-culture dish (Corning), six-well tissue culture plates (Thermo Scientific) or twelve-well culture plates (Thermo) under 5% CO_2_, 5% O_2_, and 20% N_2_ atmosphere in O_2_/CO_2_ incubator (Heracell150, Thermo) with daily medium exchange. We employed an antibiotic-free culture condition, which provides an on-going means of monitoring aseptic technique by laboratory workers. Mycoplasma infections are regularly surveyed (every quarter) by MycoAlert™ Mycoplasma Detection Kit (Lonza). The cells used in the study were all infection free. The medium included: [**1. F**] standard hESC medium supplemented with 4 ng/ml human (h) FGF2 (in house produced from yeast)^33^ and 20% v/v knockout serum replacement (KOSR, Invitrogen)^7,34^. [**2. NHSM**] knockout-DMEM (Invitrogen) with 20% KOSR, 1 mM glutamine (Invitrogen), 1% nonessential amino acids (Invitrogen), 100 μg/ml primocin (InvivoGen), 12.5 μg/ml recombinant human insulin (Sigma) and 20 ng/ml IGF1 (ProSpec) as basal medium. Cytokine and small molecules include 20 ng/ml hLIF (Millipore), 8 ng/ml hFGF2, 2 ng/ml TGFB1 (Prospec), 2 µM p38i (MAPK14 inhibitor, BIRB796, Selleckchem), 5 μM JNKi (MAPK8 inhibitor, SP600125, Tocris), 0.4 μM BMP inhibitor (LDN193189, Axon), 3 μM GSK3A inhibitor (CHIR99021, STEMCELL Technologies), and 1 μM ERK1/2i (MAP2K inhibitor, PD0325901, Selleckchem)^27^. [**3. FLB2i**] 10 ng/ml hLIF, 8 ng/mL hFGF2, 10 ng/ml BMP4 (R& D Systems), 3 μM GSK3A inhibitor, and 2 μM TGFB1 inhibitor (A83-01, Xcessbio)^19^. [**4. FL6i**] modified from NHSM by replacing TGFB1 with 1 μM TGFB1 inhibitor. Additionally, FGFR inhibitor PD173074 (STEMCELL Technologies) and JAK Inhibitor I (CAS 457081-03-7, Calbiochem) were tested in Lv-piPSC culture (Fig. 2b).

### Generation of iPSCs from porcine fetal fibroblasts with episomal vectors

Porcine fetal fibroblasts were reprogrammed with non-integrating episomal vectors according to previously described protocols^35^. 3 μg of combined three episomal plasmids (Addgene #27077, 27078 and 27079) containing human *POU5F1, SOX2, KLF4, LIN28, L-MYC* and *p53 shRNA* were electroporated with a Nucleofector II device (Lonza) and Amaxa NHDF Nucleofector kit (Lonza) into 6 × 10^5^ porcine fetal fibroblast cells derived from day 34 conceptuses^36^. The following day, the cells were placed into three 10 cm dishes previously plated with iMEF. Next day the culture medium was switched to NHSM basal medium with FGF2 and LIF (FL condition in the first dish), FLB2i medium in the second dish and FL6i medium in the third dish. 17-day post transfection, emerged independent colonies were mechanically isolated from the dishes and expanded. A total of two iPSC lines in FL, four lines in FLB2i and eight lines in FL6i conditions were established, respectively.

### Collection, production, and culture of porcine blastocysts

The collection, production and culture of porcine blastocysts was conducted by using methods described previously^37^. Porcine blastocysts were seeded on iMEFs and in pESC medium^7^, a 50:50 mixture of DMEM low glucose and Ham’s F10 medium (Thermo), supplemented with 15% fetal bovine serum (FBS; Hyclone), 2 mM glutamax, 0.1 mM ß-mercaptoethanol, 1x MEM nonessential amino acids, 1x antibiotic/antimycotic (Thermo) containing cytokines, and 20 ng/ml hFGF2 (in house)^33^. Two methods were used to generate pESCLC: plating day 7–8 of intact blastocyst and inner cell mass (ICM) isolated by immunosurgery^38^. Following 5–7 days of culture, we observed pESCLC primary colonies derived from porcine blastocysts. These pESCLC colonies were mechanically dissociated into several clumps by using pulled glass pipettes 10–15 days after seeding. Dissociated clumps were then re-seeded on fresh iMEFs, and subsequent pESCLC lines were routinely passaged via the pulled glass pipette method every 5–7 days.

### Alkaline phosphatase (AP) staining and immunocytochemistry (ICC)

AP staining was performed by the nitro blue tetrazolium/5-bromo-4-chloro-3-indolyl phosphate method (Promega). For ICC, cells were fixed in 4% w/v paraformaldehyde/PBS for 12 min, washed with PBS twice, and permeabilized with 0.1% Triton X-100 (Fisher Scientific) for 20 min After washing with PBS, the cells were incubated with 5% v/v goat or donkey serum (Sigma) in PBS for 30 min Cells were then incubated with primary antibody overnight at 4 °C. After washing, the cells were incubated with secondary antibody. Nuclei were stained with 6 ng/ml 4′,6-diamidino-2-phenylindole (DAPI) (Invitrogen). For POU5F1 staining on teratoma tissues, maximum-intensity projection of 3D montage images were obtained by using a Leica SP8 spectral confocal microscope (research.missouri.edu/mcc/leica_tcp_sp8_mp.php) to cover the large (mm size) area. Other Fluorescence images were taken under an Olympus IX70 inverted microscope at the Molecular Cytology Core at University of Missouri. Primary antibodies include: POU5F1 (1:1000 in house^39^ or 1:100; Santa Cruz Biotechnology, catalog no. sc-9081), SOX2 (1:1000; R&D Systems, MAB2018), NANOG (1:200; Abcam, ab109250), SSEA1 (1:50; Developmental Studies Hybridoma Bank [DSHB], MC-480), SSEA4 (1:50; DSHB, MC-813-70), KRT7 (1:100; DAKO, M701801-2), DESMIN (1:100; Santa Cruz Biotechnology, sc-14026), NESTIN (1:100; Abcam, ab6320), SOX17 (1:100; R&D Systems, AF1924),

### Embryoid body (EB) formation and in vitro differentiation

Epi-piPSC lines were manually isolated from the culture dish coated with iMEFs, dissociated with Accutase (STEMCELL Technologies). The dissociated cells were allowed to form EB by the hanging drop method^40^ in a droplet medium. The medium was that used for prior culture but lacked supplementary cytokines and inhibitors. After five days, the EBs were transferred to adherent, gelatin-coated tissue-culture dishes and allowed to differentiate further for 2 weeks.

### Reverse transcriptase-polymerase chain reaction (RT-PCR) analysis and quantitative PCR (qPCR)

To analyze the gene expression patterns of undifferentiated or differentiated cells, total RNA from individual samples was extracted in STAT-60 (Tel-Test Inc) according to the manufacturer’s instructions. cDNA was synthesized by using the SuperScript VILO kit (Life Technologies). PCR was performed with *Taq* DNA polymerase (PCR Master Mix, Thermo) under the following conditions: 95 ^°^C for 15 min followed by 30 amplification cycles (95 °C, 15 s; annealing temperature of specific primers, 30 s; 72 °C, 30 s) with an extension cycle at 72 °C for 1 min Quantitative PCR was performed with SYBR Green PCR kit (Life Technologies) on an ABI 7500 Real-Time PCR System (Applied Biosystem). GAPDH was used as a reference gene for this experiment. All the primers used in this experiment were shown in Supplementary Table 4.

### RNA sequencing analysis

Total RNA was extracted from the Lv-piPSC, Epi-piPSCs and pESCLC lines cultured in respective conditions by using STAT-60. The quantitation and quality control of RNA from all samples was performed on a Fragment Analyzer (Advanced Analytical), and cDNA libraries were constructed by standard methods (Illumina TruSeq mRNA stranded kit) with index adapters (Illumina TruSeq indexes). The RNA-Seq data were generated on the Illumina (HiSeq 2500 instrument) platform by 50 bp paired-end sequencing. The sequencing reads were aligned onto Sscrofa11.1 reference genome by TopHat v2.1.0 software^41^ . The mapped reads were transformed into the count matrix with default parameters by HTSeq v0.8.0 software^42^, followed by normalization with DESeq v2 software^43^. Differentially expressed genes (DEGs) were identified by means of the same software, DESeq2, based on negative binomial generalized linear models. GO enrichment analysis of the DEGs was performed by using PANTHER (www.pantherdb.org/)^44^. For the visualization of enriched GO terms in ranked lists of DEGs and their clustering, all heatmaps were generated by the Heatmapper (www.pantherdb.org/)^45^ with log2 transformed values.

### Teratoma formation

20 million cells of the Epi-piPSC, Lv-piPSC and Epi-hESC lines obtained by Dispase dispersion of attached colonies (1 mg/mL, STEMCELL Technologies) were centrifuged (200×*g* for 5 min), resuspended in 0.2 mL of original culture medium, and chilled on ice before mixing with 0.14 ml of Matrigel (Corning) supplemented with ROCKi, Y-27632 (final 10 µM). The solution was loaded into a 1-mL syringe (Becton Dickinson) and injected subcutaneously into 8 to 12-week-old non-obese diabetic SCID-γ mice (for all except Lv-piPSC-F, Jackson Laboratories) or CD-1 nude mice (for Lv-piPSC-F, Charles River) on their flanks through 22-gauge needles (10 million cells of the former and 1 million for the latter per site). The tumors were dissected out after euthanizing the mice and fixed in 10% (v/v) neutral buffered formalin overnight. Paraffin-embedded tissue was sectioned and then stained with H&E. All animal experiments were approved by the University of Missouri Institutional Animal Care and Use Committee under Protocols 7170 and 8053.

### Statistical analysis

For qPCR results, the mRNA abundance levels of a target gene were normalized to the internal control gene, *GAPDH*, and the relative expression value were determined by comparative threshold cycle method^46^. Data were analyzed by using the relative expression tool REST 2009 version 2.0.13^47^. DESeq2 was used to detect significantly differentially expressed genes in the RNAseq analysis^43^. For cell doubling time, the comparison was performed by permutation tests for the differences between groups of growth curves from the R project for statistical computing^48^.

## Results

### Transition of primed type porcine iPSC to naïve-like pluripotency state

**Fig. 1:**
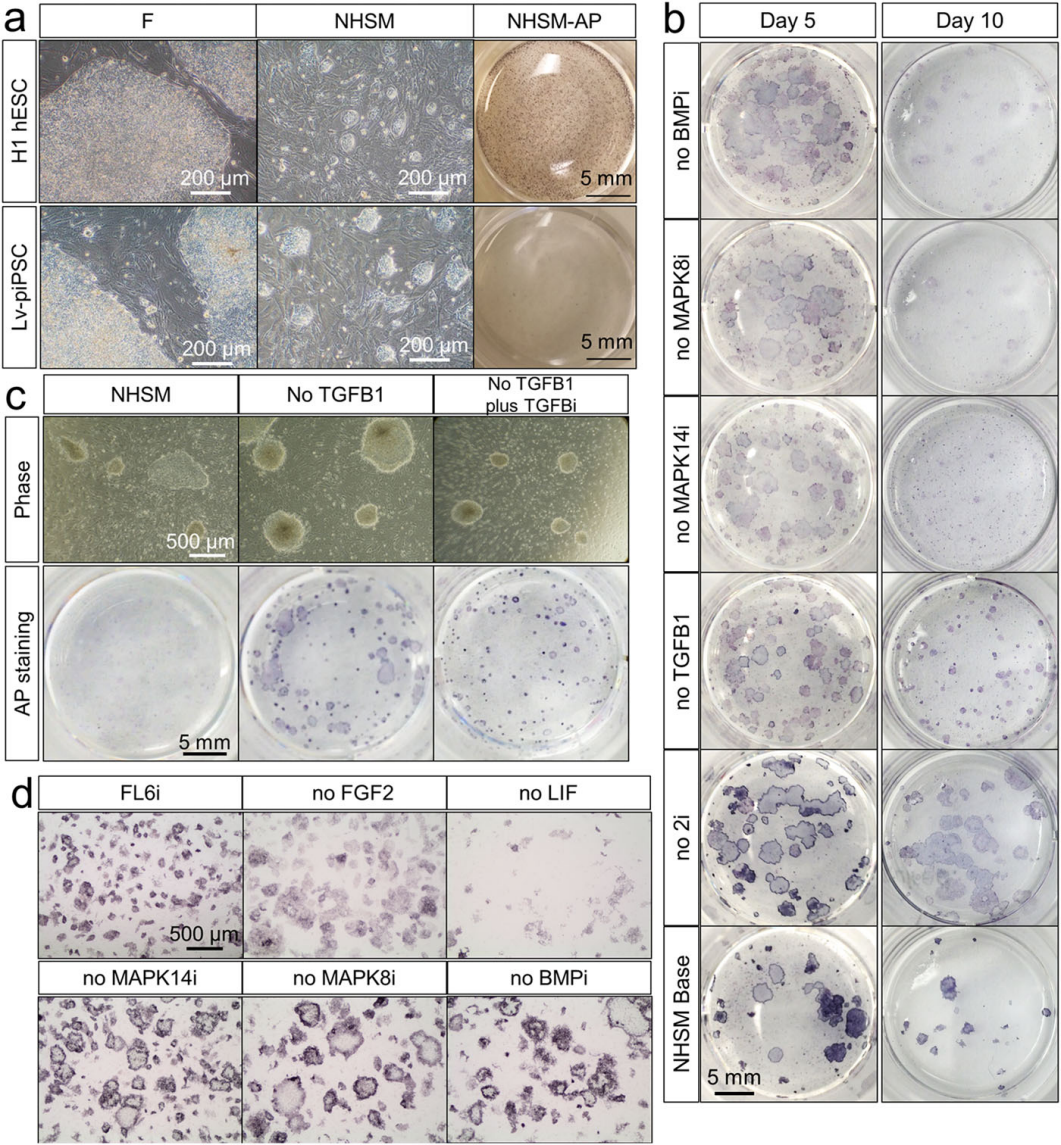
. **a** Live cell images of H1 hESC and Lv-piPSC under F and NHSM conditions with bars, 200 µm and AP staining images in NHSM condition with bars, 5 mm. **b** AP staining images of LV-piPSC following removal of individual components from NHSM. AP staining was performed at Day 5 and Day 10 of culture under each condition with bar, 5 mm. **c** Live cell (bar, 500 µm) and AP staining (bar, 5 mm) images of Lv-piPSC under NHSM, NHSM without TGFB1 and NHSM without TGFB1, plus TGFB inhibitor (TGFBi) conditions. **d** AP staining images of Lv-piPSC under FL6i condition and FL6i in absence of respective components (bar, 500 µm)

We examined whether the NHSM^27^ could be used for culturing the primed type lentiviral reprogrammed piPSC (Lv-piPSC) ID6 line that has been maintained in DMEM/F12 medium supplemented with FGF2 (termed as F; Supplementary Table 1b)^34^. The human ESC (hESC) line (WA01/H1) and Lv-piPSC were adapted to NHSM on a feeder layer of irradiated mouse embryonic fibroblasts (iMEF). After 10-day of culture, colonies of both H1 and Lv-piPSC in F began to exhibit dome-shaped morphologies that resembled those of mouse naïve type ESC (Fig. 1a). However, unlike the hESC, Lv-piPSC were negative for alkaline phosphatase (AP), indicating a loss of pluripotency (Fig. 1a). Primed type Lv-piPSC were then tested for growth on NHSM with the following modifications to identify which component in that medium had caused negative consequences of maintaining AP activity: 1. NHSM with FGF2 and LIF but without inhibitors of MAP2K, MAPK8, MAPK14, GSK3A, and TGFB1; 2. NHSM without the two inhibitors of MAP2K and GSK3A; 3. NHSM without TGFB1; 4. NHSM without inhibitor of MAPK14; 5. NHSM without inhibitor of MAPK8; 6. NHSM without inhibitor of BMP. (Supplementary Table 1a). Surprisingly, after 10-day culture, only condition 3 (NHSM without TGFB1) provided dome-shaped colonies that were AP-positive (Fig. 1b), suggesting the presence of TGFB1 that caused the loss of AP activity in Lv-piPSC. By replacing TGFB1 with its inhibitor, the proportion of compact dome-shaped, AP-positive colonies relative to flattened colonies increased nine-fold (Ratio in no TGFB1, 1.08; ratio with TGFB1 inhibitor, 9.92) (Fig. 1c). As a result, we developed an optimized medium containing a combination of FGF2, LIF, inhibitors of TGFB1, MAP2K1, MAPK8, MAPK14, GSK3A and BMP (termed FL6i medium) to convert the primed type Lv-piPSC-F into naïve type cells. To ratify the whether all the components (FGF2, LIF, inhibitors of MAPK8, MAPK14, and BMP) were required for maintaining the AP-positive/naïve type colony morphology, each compound was sequentially omitted from the FL6i medium. Only cells on the complete medium were converted from the primed to naïve-like state (Fig. 1d).

### Characterizations of Lv-piPSC in FL6i medium

**Fig. 2:**
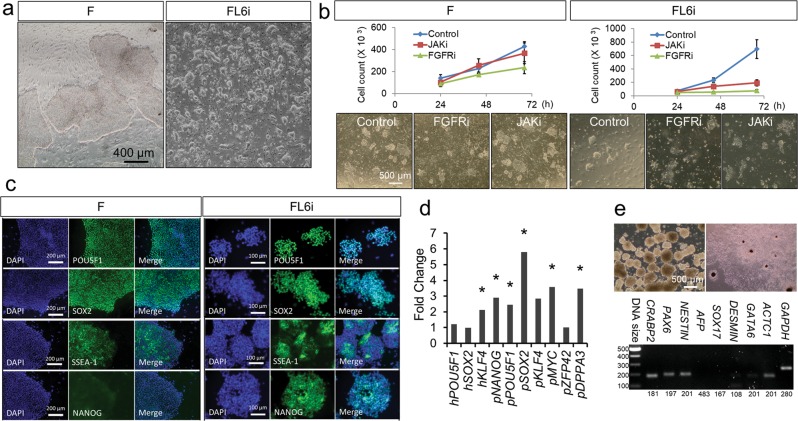
**a** Live cell images of Lv-piPSC under F and FL6i conditions with bar, 400 µm. **b** Cell proliferation curve (upper panel) and live cell images (bottom panel, bar, 500 µm) of Lv-piPSC treated with 1 µM PD173074 (FGFRi), 0.6 µM JAK Inhibitor I (JAKi) or control in F and FL6i conditions. **c** ICC of pluripotency markers, POU5F1, SOX2, SSEA-1 and NANOG, in Lv-piPSC under F and FL6i conditions with bars, 200 and 100 µm. **d** The relative expression level of pluripotency genes in Lv-piPSC under FL6i conditions determined by qPCR. The expression value of each gene in Lv-piPSC under F condition was arbitrarily set to 1. Asterisk symbol (*) represents significant differences (*p* < 0.05) in gene expression level between Lv-piPSC under F and FL6i conditions. The experiments were replicated three times. **e** The formation of embryoid bodies (EB, left) and adherent outgrowths of EB (right) derived from Lv-piPSC-FL6i (upper panels, bar, 500 µm) and the expression of differentiation markers representing three germ layers (*CRABP2, PAX6, NESTIN*: ectoderm, *AFP, SOX17, GATA6*: endoderm, *DESMIN, ACTC1*: mesoderm) determined by reverse transcription PCR (bottom panel). The product sizes in bp are listed

The naïve like Lv-piPSC cultured in FL6i (Lv-piPSC-FL6i) not only resembled mouse naïve-type ESC (Fig. 2a), but were dispersed into single cells for passaging and had shortened cell doubling time (from 17 h in F to 13.5 h in FL6i, *p* = 0.017). However unlike mouse ESC, they appeared to rely on both FGF/ACTIVIN/NODAL and LIF/JAK/STAT signaling for cell self-renewal (Fig. 2b). They were positive for the pluripotency markers SSEA1, NANOG, POU5F1 and SOX2 (Fig. 2c), and the expressions of several pluripotency-related endogenous genes (*pNANOG, pPOU5F1, pSOX2, pMYC, pDPPA3*) were significantly increased (Fig. 2d). Despite this upregulation of endogenous genes, the expression of the ectopic genes (*hPOU5F1 hSOX2, hKLF4*) used initially to drive induced pluripotency remained relatively unchanged under the new culture conditions (Fig. 2d). The Lv-piPSC-FL6i formed embryoid bodies and underwent spontaneous differentiation. The differentiated cells were positive for ectoderm and mesoderm markers, but they failed to express endoderm markers (Fig. 2e).

### Derivation of episomal plasmid-mediated piPSC (Epi-piPSC)

**Fig. 3:**
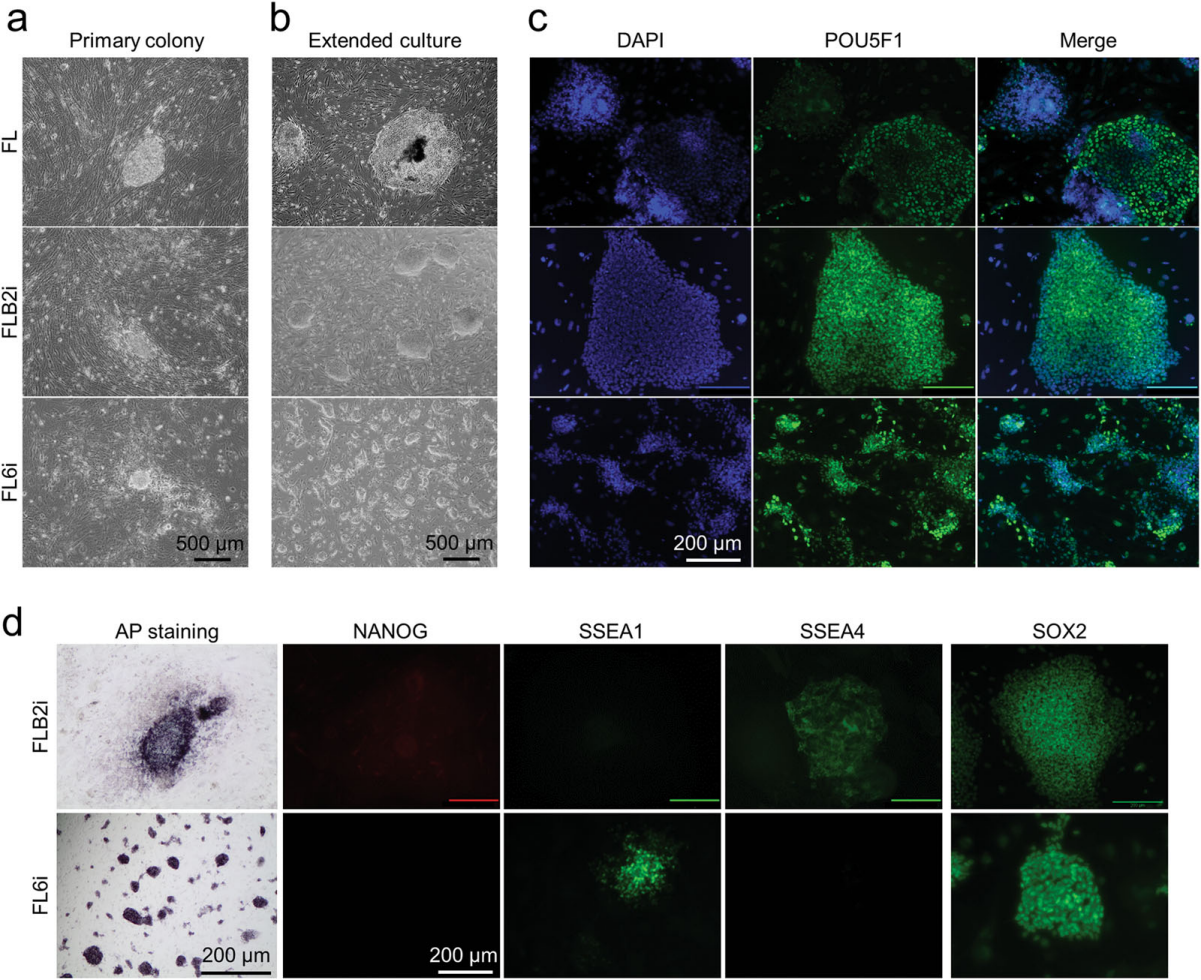
Live cell images of ‘primary colonies’ (**a**) and established colonies after 40 days of “extended culture” (**b**) of Epi-piPSC under the three culture conditions. **c** ICC of POU5F1 of Epi-piPSC under the three conditions after 40 days of extended culture. **d** AP staining and ICC of NANOG, SSEA1, SSEA4 and SOX2 of Epi-piPSC lines established in FLB2i and FL6i conditions

We next examined whether FL6i condition could be used to generate transgene-free piPSC by using non-integrating episomal plasmids^35,49^. Porcine fetal fibroblast cells were transiently transfected with the plasmids^35^ and plated onto iMEF feeder layers under three culture conditions: 1, NHSM basal medium with FGF2 and LIF (FL); 2, a previously reported medium claimed to generate an intermediate pluripotent state in piPSC^19^ (FLB2i); 3, the newly developed FL6i described above. Two to three weeks after the transfection, primary colonies with flat morphologies were observed in FL, mounded colonies in FLB2i, and small dome-shaped colonies in FL6i (Fig. 3a). Individual colonies were manually picked and propagated by passaging under the three respective conditions (Fig. 3b), whereas colonies from FLB2i and FL6i conditions demonstrated homogenous POU5F1 expression and were undifferentiated (Fig. 3c). Colonies in FL condition were comprised largely of epithelial-like cells (Fig. 3b) with much reduced POU5F1 expression (Fig. 3c). As culture on FL was extended, the cells stopped growing and no iPSC line was established. However, it was possible to generate multiple piPSC in FLB2i (Epi-piPSC-FLB2i) and FL6i conditions (Epi-piPSC-FL6i). All were positive for AP and expressed POU5F1 and SOX2, but all stained negatively for NANOG (Fig. 3d). While Epi-piPSC-FL6i expressed SSEA1, the Epi-piPSC-FLB2i cells were SSEA4 positive (Fig. 3d).

**Fig. 4:**
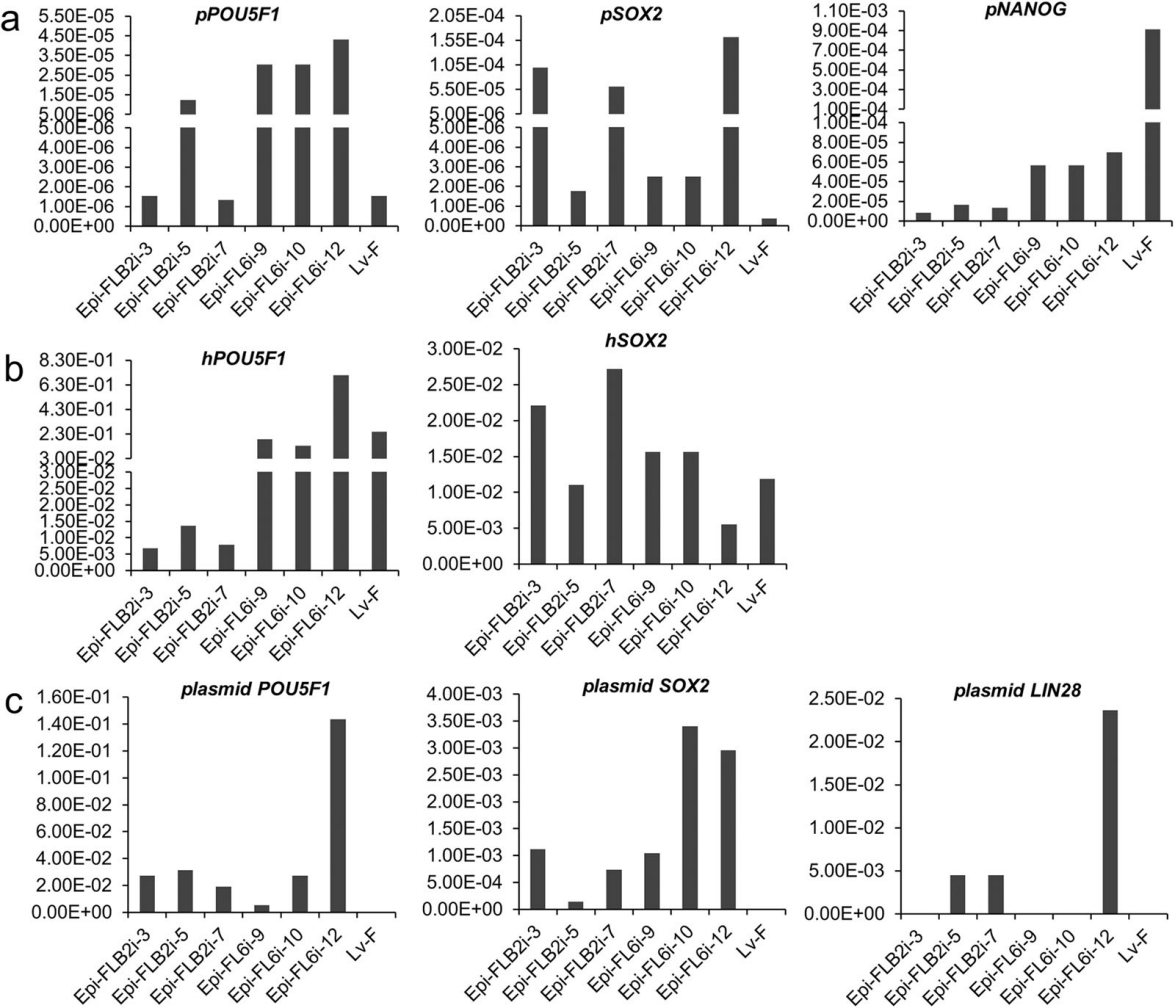
. Quantitative comparison of endogenous (**a**) and exogenous (**b**, **c**) pluripotency marker expressions in Epi-piPSC-FLB2i, Epi-piPSC-FL6i and Lv-piPSC-F linesThe relative expression levels of the target genes were normalized against the expression level of *GAPDH* from the same sample (set as 1). Expression of the endogenous porcine genes (*pPOU5F1*, *pSOX2*, *pNANOG* in (**a**) and exogenous human genes (*hPOU5F1*, *hSOX2* in (**b**) were determined by species specific primers for respective coding sequences. Expression values from the episomal plasmids (plasmid *POU5F1*, plasmid *SOX2*, plasmid *LIN28* in (**c**) were quantified by paired primers of gene specific and the plasmid backbone. The primer information is shown in Supplementary Table 4

To examine whether any of the reprogramming genes and the plasmids persisted in the established Epi-iPSC, six lines of Epi-iPSC (three from FLB2i, three from FL6i) were randomly selected from each culture condition after 80 days of continuous culture (15–20 passages). Specific PCR primers were designed to provide a comparison between transcript levels from endogenous pluripotency genes and those from the exogenous reprogramming genes. Although each reprogrammed cell line was weakly positive for expression of *pPOU5F1* and *pSOX2*, values were quite variable. The levels of *pNANOG* transcripts were higher in FL6i than in FLB2i. As observed with the Lv-line in F, all six cell lines robustly expressed *hPOU5F1* and *hSOX2*, (Fig. 4b). Detection of *hPOU5F1, hSOX2*, and *hLIN28* with the vector backbone DNA (performed with gene specific primer and the vector-specific primer) (Fig. 4c; *plasmid POU5F1, - SOX2, - LIN28*), suggests that the episomal-plasmids had persisted in the cells, possibly through integration into host DNA.

### Teratoma formation from Epi-piPSC generated under different culture conditions

**Fig. 5:**
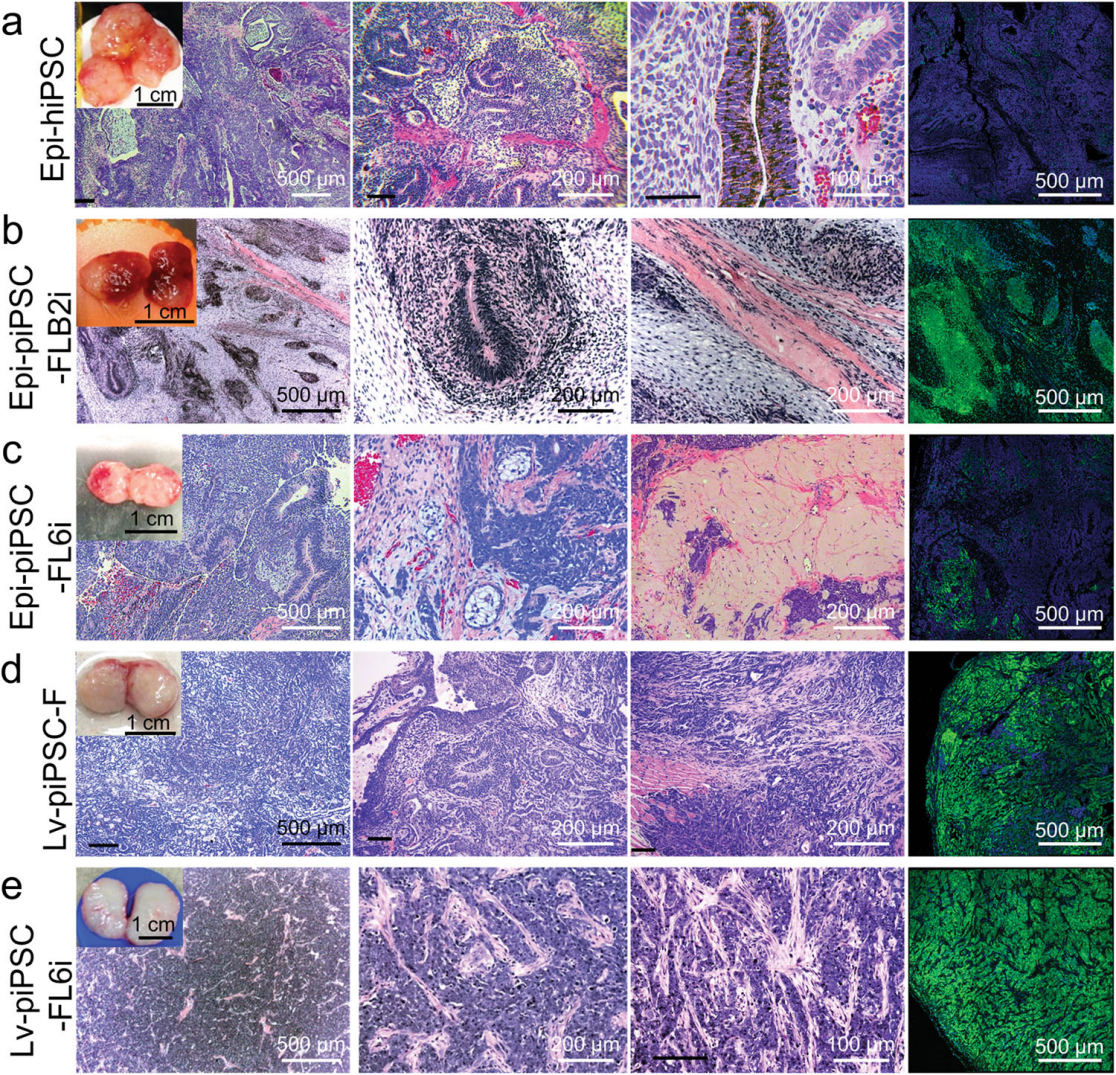
Tumor tissues generated from different porcine iPSC in immunodeficient mice. Tissue generated from transgene free hiPSC, Epi-hiPSC (**a**), Epi-piPSC-FLB2i (**b**), Epi-iPSC-FL6i (**c**), Lv-piPSC-F (**d**), Lv-piPSC-FL6i (**e**). Inserted images in the left panels show the gross morphology of the tumors immediately after collection from the host mice, bars 1 cm. The panels in rows 1–3 illustrate H&E staining of the histological section of the tumors, bars, 500 µm, 200 µm or 100 µm. Far right panels show the ICC for POU5F1 in respective tumor tissues with bars, 500 µm

To evaluate the pluripotency of the newly developed Epi-piPSC and compare their ability in generating differentiated cell types, teratoma studies were conducted in immunodeficient mice. Teratomas from two Epi-piPSC-FLB2i and three Epi-piPSC-FL6i sublines were compared with those from three well characterized iPSC lines (a transgene free human iPSC, Epi-hiPSC and two Lv reporgrammed piPSC (Lv-piPSC-F) and Lv-piPSC-FL6i) (Supplementary Table 3). All were able to produce encapsulated solid tumors that were removed between days 38~ 90 after the injection. As expected, the Epi-hiPSC teratoma (Fig. 5a) included tissue representative of ectoderm, endoderm, and mesoderm, and POU5F1 expression was almost absent (Fig. 5a). The tumors produced by Epi-piPSC-FLB2i, Epi-piPSC-FL6i and Epi-hiPSC were softer than the Lv-piPSC teratomas and contained hemorrhagic areas when they were examined in cross-section (Figs. 5a–c). The tumor derived from Epi-piPSC-FLB2i lacked apparent endoderm derivatives but contained ectoderm and mesoderm (Fig. 5b). The tissue contained islands of POU5F1 expression between more differentiated areas (Fig. 5b). However, the tumors from Epi-piPSC-FL6i had overall lower POU5F1 expression than those from the Epi-piPSC-FLB2i and also contained diverse tissue types representative of ectoderm, endoderm and mesoderm (Fig. 5c). By contrast, the Lv-piPSC-F and Lv-piPSC-FL6i lines formed homogeneous tumors when viewed in cross section (Figs. 5d, e). The tumors were also largely undifferentiated, with a majority of POU5F1-positive cells. Nevertheless, islands of ectoderm, mesoderm and endoderm were scattered throughout the tissue from the Lv-piPSC-F lines (Fig. 5d). Tumors from Lv-piPSC-FL6i lines had a largely undifferentiated morphology and were POU5F1-positive throughout, except in diffused eosinophilic cells of connective tissue (Fig. 5e).

### Attempts to generate pESC with the modified media

First, we attempted to generate primed-type pESC in standard FGF2-supplemented medium, i.e., pESM conditions^7^ (termed F) from either day 7–8 intact blastocysts or immunosurgery isolated ICM^38^. Both approaches yielded similar types of outgrowth at similar efficiencies. Higher outgrowth efficiency was achieved with embryos that were beginning to hatch (Supplementary Table 2). The all outgrowths were similar in morphology and were positive for AP.

**Fig. 6:**
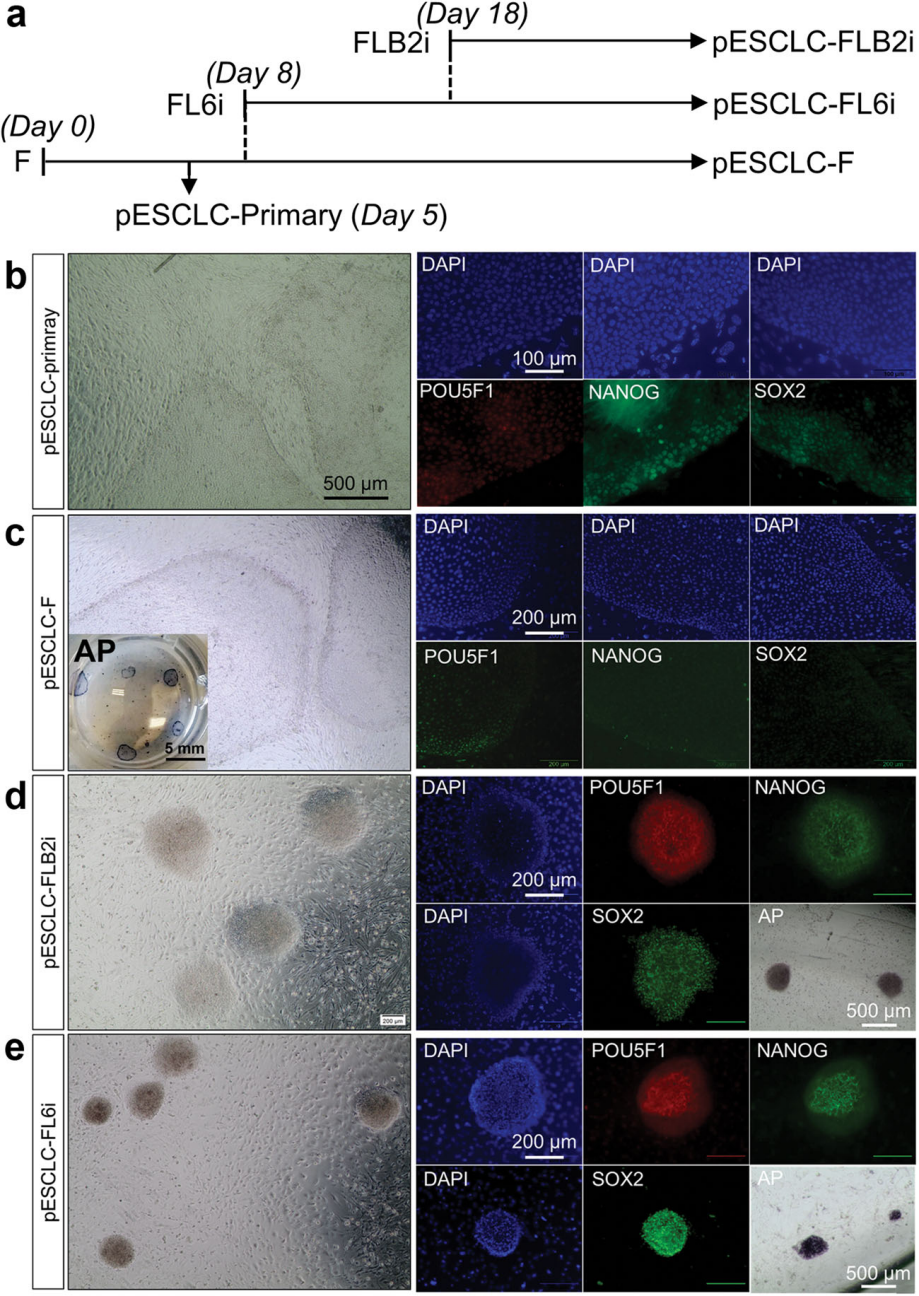
Porcine ESC like cell (pESCLC) generation under F, FLB2i and FL6i conditions. **a** Schematic diagram illustration how the pESCLC were generated under F, FLB2i and FL6i conditions. Representative live cell image and POU5F1, NANOG and SOX2 ICC plus AP staining for cell colonies generated under the different culture conditions: pESCLC-primary (**b**), pESCLC-F (**c**), pESCLC-FLB2i (**d**) and pESCLC-FL6i (**e**). Bars are 100 µm in (**b**) 200 µm in (**c**-**e**). **f** Heatmap analysis of 19 porcine genes whose expressions are linked to pluripotency in the ESC-like cells generated under different conditions

Attempts were also made to generate pESC under FL6i (from both blastocysts and ICM, Supplementary Table 2) and FLB2i (from blastocysts only) culture conditions. Fewer outgrowths were observed than under F, and all the attached colonies were unstable, i.e. quickly differentiated and stopped proliferating within two weeks (Supplementary Fig. 1b, c). As an alternative to using these two defined media, outgrowths were generated under F (termed pESC like cells, pESCLC-primary; Figs. 6a, b, Supplementary Fig. 1a). These outgrowths were manually passaged and maintained in F (named pESCLC-F)^7^, Some of the primary outgrowths were also switched to FLB2i and FL6i conditions (Fig. 6a). Further growth on FLB2i failed, but, in FL6i conditions, small foci (50–80 µm in diameter) became visible within the colonies (Supplementary Fig. 1d) and adopted dome-shaped morphologies (Fig. 6e, Supplementary Fig. 1f). From these small foci, we generated a continuously proliferating cell line (named pESCLC-FL6i), whose morphologies resembled the earlier described Epi-piPSC-FL6i. After 10-day culture under FL6i, some of the colonies were sub-cultured into FLB2i conditions, and they continued to proliferate, forming mounded colonies resembling the Epi-iPSC-FLB2i colonies described earlier (Fig. 6d, Supplementary Fig. 1e). Thenceforth, all three lines, pESCLC-F, pESCLC-FLB2i and pESCLC-FL6i were manually passaged every 4–7 days on iMEF feeders. Each cell line proliferated for at least 15 passages over a ~2-month period.

All three pESCLC lines were examined for expression of POU5F1, NANOG, SOX2 and AP at 16, ~34, and ~46 days (passage numbers 2, 6, and 9, respectively), and compared with initial blastocyst outgrowths (pESCLC-primary) that had been collected at day 8~10 (Figs. 6b–e). The pESCLC-primary outgrowths expressed all three pluripotent markers examined (Fig. 6b). However, the cell cultures continued in F (pESCLC-F) showed increasingly lower SOX2 and POU5F1 expressions although NANOG and AP activity remained relatively constant (Fig. 6c). When the pESCLC were maintained in FLB2i (Fig. 6d) or FL6i (Fig. 6e), the marker expressions persisted without obvious decline.

### Comparative gene expression analysis of cell lines

Transcriptome profiles of the three putative pESC (pESCLC-F, pESCLC-FLB2i, pESCLC-FL6i), the three piPSC lines (Lv-piPSC-F, Epi-piPSC-FLB2i, Epi-piPSC-FL6i), each in duplicate representing different passage number, and two primary blastocyst outgrowths were compared by using RNAseq. A heatmap analysis of 19 known porcine genes whose expression has been linked to pluripotency^19^ was then generated (Fig. 6f). The primary blastocyst outgrowths (pESCLC-primary) had the most consistent upregulation of expression of these genes, including *POU5F1* and *NANOG*, relative to any of the cell lines. One other feature of these primary cultures was relatively low *SOX2*, *ESRRB*, and *KLF4* expression. However subsequent culture of these pESCLC cells in either FLB2i or FL6i medium led to a down regulation of the majority of these pluripotency genes, including *POU5F1* and *NANOG* but not *SOX2*. By contrast, continued culture under F, had a more minor effect, although some key genes, e.g., *POU5F1, SOX2*, and *ESRRB* became expressed at lower levels than in their primary progenitors. Of the remaining cell lines, the lentivirus-transformed cells (Lv-piPSC) cultured on F medium clustered most closely to the blastocyst outgrowths, followed by the episomally-transformed cells (Epi-piPSC), cultured on FL6i medium, although in both there was marked down regulation of *TET2* and *TET3* genes (Fig. 6f).

**Fig. 7:**
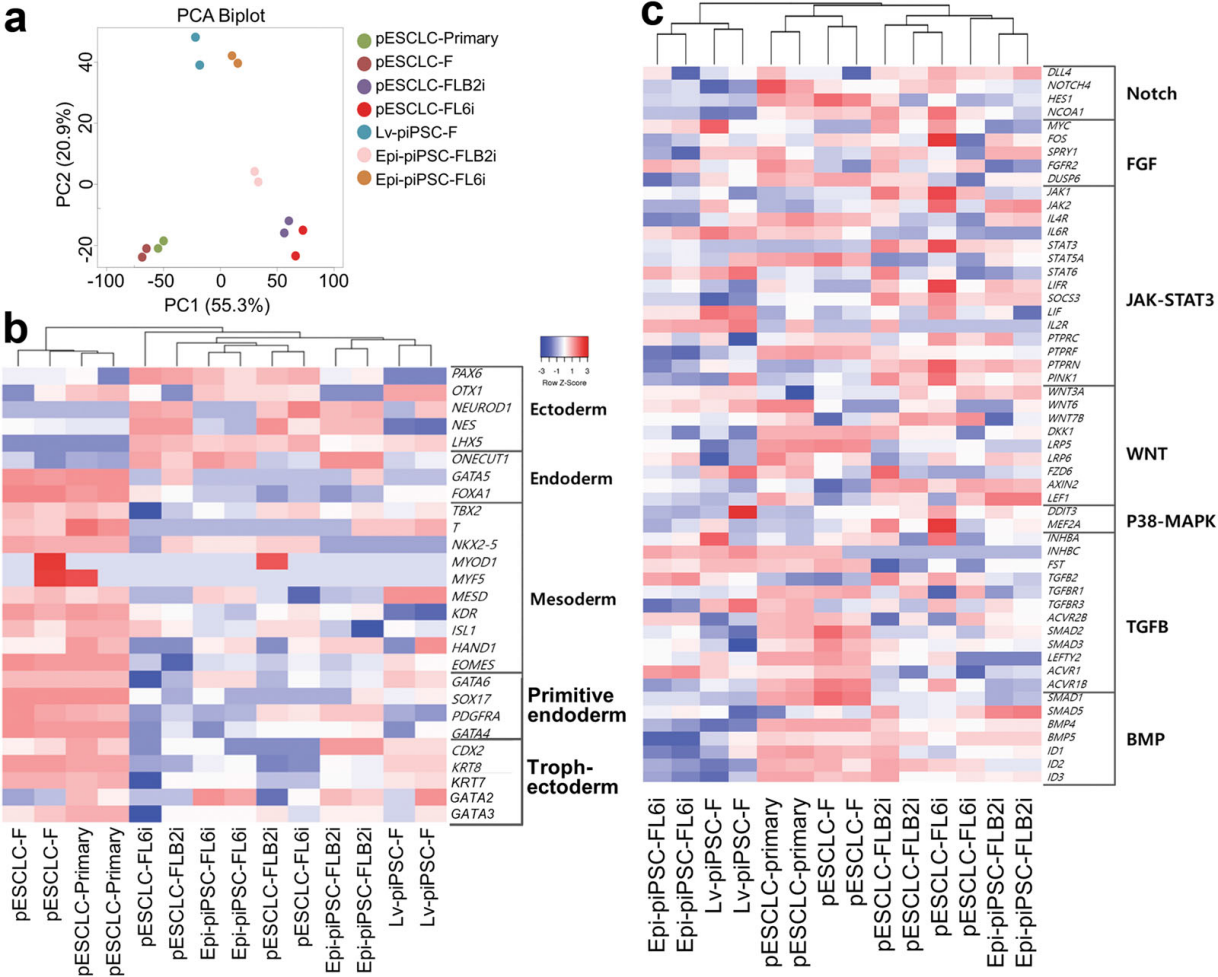
Comparative gene expression analysis of three piPSC (Lv-piPSC, Epi-piPSC-FLB2i and Epi-piPSC-FL6i) and three pESCLC (pESCLC-F, pESCLC-FLB2i, pESCLC-FL6i) lines and primary blastocyst outgrowth (pESCLC-primary). **a** PCA based on RNAseq data from the seven cell samples in duplicate. **b** Heatmap of 27 genes linked to early embryonic differentiation to ectoderm, mesoderm, endoderm, primitive endoderm and trophectoderm from duplicated seven cell samples. **c** Heatmap of 54 genes of seven major signaling pathways (Notch, FGF, JAK-STAT3, WNT, p38 (MAPK14), TGFB and BMP) associated genes in each cell types

A principle component analysis (PCA) based on RNAseq data showed distinct distributions according to how the cells were created (pESCLC vs. piPSC) and culture conditions (Fig. 7a). The pESCLC lines were clearly separated according to the culture conditions, i.e., F vs. FL6i/FLB2i, on the PC1 dimension while Epi-piPSC (FL6i vs. FLB2i) lines showed differences primarily by PC2 values. Although the Lv-piPSC was grown in F, it showed a close relationship with Epi-piPSC cultured under FL6i conditions.

We also compared the cell lines by hierarchical clustering of differentially expressed genes (DEGs; fold change ≥ 3, adjusted *p* ≤ 0.05) (Supplementary Fig. 2 and Supplementary Table 5). Here the culture conditions appeared to provide the greatest discrimination between groupings. Only 242 DEGs out of 17,650 genes analyzed distinguished the pESCLC-primary and pESCLC-F. Far larger numbers of DEGs separated pESC-primary from both pESC-FLB2i (1,945 DEGs from 18,085) and pESCLC-primary vs pESCLC-FL6i (2,058 DEGs from 18,110), which is consistent with the PCA analysis (Fig. 7a).

Gene Ontology (GO) enrichment analysis was used to gain insights into the degree to which the different cell lines showed signs of differentiation. (Supplementary Fig. 3). The top 10 categories in the overall GO list for all cell lines included developmental process, ectoderm development, cell differentiation, mesoderm development, and cell signaling pathways. Based on this outcome, we analyzed the relative expressions of 27 genes linked to early embryonic differentiation^50,51^ (Fig. 7b). The pESCLC-primary and pESCLC-F group were again widely separated from the others, largely due to the high expression of genes of all the principal early embryonic lineages with the exception of ectoderm. The cell line that showed the lowest expression of differentiation markers was the Epi-piPSC-FL6i. In fact, differentiation markers for mesoderm and endoderm were relatively low in all FLB2i and FL6i cultures and in the Lv-piPSC-F, although there appeared to be some up-regulation of ectoderm markers.

We next focused on DEGs linked to seven major signaling pathways (Fig. 7c)^51,52^. Genes associated with TGFB and BMP signaling were upregulated in pESCLC-primary and pESCLC-F group, whereas low Notch and BMP signaling distinguished the Lv-piPSC and the Epi-piPSC grown on FL6i medium from the rest (Fig. 7c).

## Discussion

Here we have demonstrated that primed type Lv-piPSC transition to naïve-like pluripotency under the FL6i conditions. One major change made in FL6i relative to the original NHSM^27^ is the replacement of TGFB1 with a TGFB inhibitor, SB431542. TGFB1 activates ERK/MAPK signaling by SHC1 (SHC-transforming protein 1) phosphorylation as well as via SMAD2/3 signaling, both of which are crucial for the maintenance of pluripotency in epiblast-type human PSC^53,54^. In contrast, TGFB1 is dispensable for the maintenance of naïve state mouse ESC^55^, although inhibition of TGFB signaling improves iPSC reprogramming efficiency^56^ and helps prevent rat PSC from spontaneously differentiating^57,58^. The role of TGFB signaling in maintaining porcine pluripotency is less clear. TGFB/ACTIVIN/NODAL signaling is required for self-renewal of primed-type piPSC^59^ and putative porcine epiblast stem cells^6^. On the other hand, the TGFB/ACTIVIN inhibitor, SB431542, used in our experiments has been employed in a number of other protocols for generating piPSC^10,60,61^. Interestingly, all the piPSC generated in presence of SB431542 had morphologies similar to naïve state mouse ESC, whereas piPSC and pESCLC dependent on TGFB were in the primed state. Thus, like human and mouse, primed state pluripotency in porcine cells is probably dependent on the activation of SMAD2^62,63^, while naïve state pluripotency requires inhibition of TGFB signaling^64^.

In contrast to TGFB1, BMP4 signaling favors SMAD1/5/8 signaling, which, in turn, causes the activation of inhibitors of DNA binding proteins (IDs). In mouse naïve-type ESC, for example, BMP4 suppresses differentiation and, in combination with LIF, sustains self-renewal^65^. BMP4 inhibition also assists the stabilization of human ESCs in what is sometimes considered to be a ground state, i.e. naïve-type^26^. The NHSM also contains BMP inhibitor as an optional component to boost human naïve pluripotency^27^. FL6i, described in this paper, contains both inhibitors of TGFB1 and BMP. Under such conditions the Lv-piPSC demonstrated more compact, dome-shaped colony morphology, a shortened cell doubling time, increased AP activity, and tolerance to single cell passage, which are hallmarks of mouse, naïve state, PSC. FL6i also enhanced the expression of endogenous pluripotent genes, including *NANOG*, *POU5F1*, *SOX2*, *MYC*, and *ZFP42*, although the expression of the ectopic reprogramming genes remained high (Fig. 2d). We inferred that FL6i promoted porcine pluripotency and might, therefore, support the derivation of piPSC without accompanying transgene integration.

Accordingly, we then attempted to generate piPSC from porcine embryonic fibroblasts in FL6i medium as well as in two other culture conditions via non-integrating episomal vectors^35^. The control medium containing FGF2 and LIF but no small molecule additions, did yield primary colonies (Epi-piPSC-F), although it proved impossible to derive stable cell lines from these starter colonies over extended culture (Figs. 3a–c) With both FL6i and FLB2i conditions, colonies with a naïve-type phenotypes were readily generated and propagated successfully over several weeks without either apparent loss of self-renewal or signs of differentiation (Fig. 3). However, none of the cell lines established were transgene-free at this stage (Fig. 4), although endogenous pluripotency genes were upregulated (Fig. 4) and expression of early differentiation genes low (Fig. 7b). FL6i culture conditions, in particular, provided piPSC superior to those cultured under FLB2i conditions with upregulation of the 19-gene cohort of pluripotency markers (Fig. 6f) and their ability to generate well differentiated tumors largely lacking expression of POU5F1 (Figs. 5b, c). A recent study employed the same episomal plasmid approach as ours with 2i plus FGF2 condition^14^. Like ours, the generated piPSC showed presence of vector DNA, but integration-free piPSC could be selected from the low passage cells by further subcloning. We conclude that the piPSC generated under FL6i conditions have considerable promise.

Reprogramming via ectopic transcription factors, as illustrated here with episomal plasmids has been categorized into two phases^66^. First, a stochastic phase in which expression of transgenes triggers random epigenetic events that cause global changes of the somatic epigenome into an ESC-like status^67^. Possibly, reprogramming in the basal FL medium initiated these events but did not allow progression to the second, deterministic phase, in which the presence of inhibitors, is believed to enhance the expression of endogenous pluripotency genes needed to sustain a stable pluripotency state^66–68^.

In the third part of this study, we attempted to generate pESC under different culture conditions. Efforts to obtain pESC directly from blastocysts under FLB2i and FL6i conditions failed. By contrast, basal FGF2 medium allowed initial attachment and colony formation, while subsequent culture under FLB2i and FL6i conditions permitted cell lines indistinguishable in appearance to naïve type piPSC to be isolated. Although the initial blastocyst outgrowths demonstrated relatively robust expression of genes associated with the pluripotent state (Fig. 6f) they also had higher level expression of early differentiation markers representing mesoderm, endoderm, primitive endoderm and extraembryonic lineages (Fig. 7b). Although these differentiation markers were downregulated in pESCLC under FLB2i and FL6i conditions, there remained a higher level of ectoderm markers (Fig. 7b) and relatively weak expression of the cohort of pluripotency genes (Fig. 6f). We conclude that these cell lines are still less than ideal as bona fide ESC.

Stable bovine ESC lines were recently derived from preimplantation blastocysts by employing supplementary FGF2 and a WNT signaling pathway inhibitor^69^. A possible explanation for why FGF and WNT signaling inhibition in combination capture bovine pluripotency is their ability to regulate the balance of lineage specification between mesoendoderm and ectoderm^70–72^. It appears that this equilibrium is more difficult to achieve in pESC. While FLB2i and FL6i conditions appear capable of blocking differentiation towards mesoendoderm, the cells seemed more prone to differentiate into ectoderm (Fig. 7b). Additionally, it is apparent that TGFB and BMP signaling must also be blocked in order to advance towards a pluripotent state in pESC. For example, there was a significant downregulation of the gene markers representing these two pathways in our Epi-piPSC-FL6i and Lv-piPSC but an upregulation under other FGF2 conditions (Fig. 7c). These observations may lead to optimized culture conditions that allow derivation of stable pESC in the near future.

In conclusion, we report culture conditions of FL6i that support enhanced naïve-state pluripotency in Epi-piPSC. In particular, blocking the TGFB and BMP signaling pathways was an essential issue in reaching this goal. Although the final goal of generating transgene-free piPSC and authentic pESC by using the FL6i medium has not been achieved, the cell lines created point the way to establishing protocols that will finally allow stable, fully pluripotent porcine ESC and iPSC to be generated for potential biomedical and agricultural purposes.

### Deposition of transcriptome data

The datasets analyzed during the current study are available in the Gene Expression Omnibus (GEO) repository, GSE126150.

## Supplementary information

Supplementary Fig 1

Supplementary Fig 2

Supplementary Fig 3

Supplementary Table 5

Supplemental Material File #1

Supplemental Material File #2
