# Supplementary figures and images for: A six-inhibitor culture medium for improving naïve-type pluripotency of porcine pluripotent stem cells
Source: Cell Death Discov. 2019 Jun 17;5:104. doi: 10.1038/s41420-019-0184-4 (PMC6579764; doi:10.1038/s41420-019-0184-4)

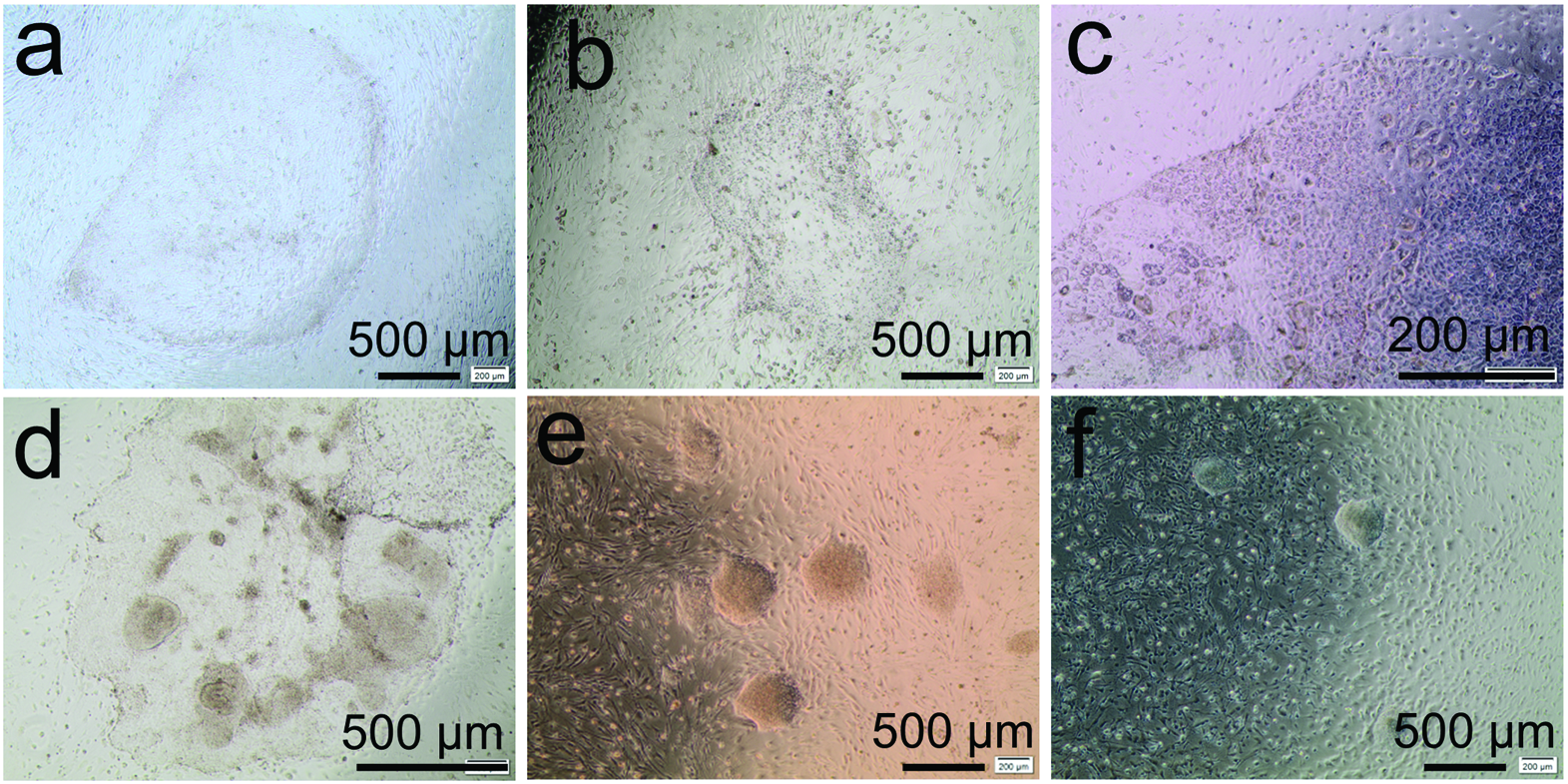

Supplement: Supplementary file 1 — Supplementary Fig 1 [file 41420_2019_184_MOESM1_ESM.tif]

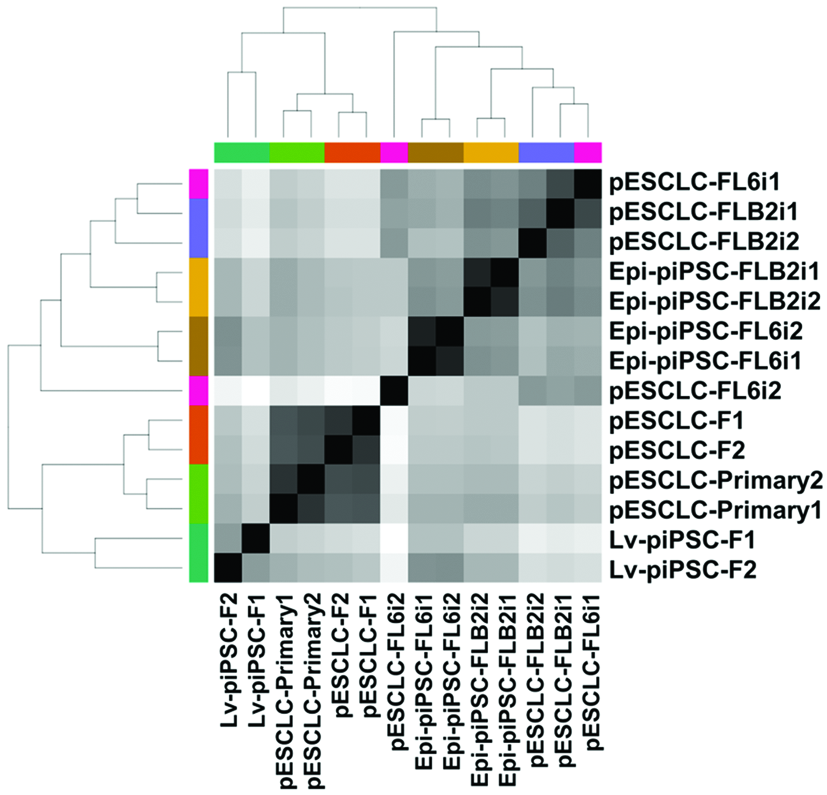

Supplement: Supplementary file 2 — Supplementary Fig 2 [file 41420_2019_184_MOESM2_ESM.tif]

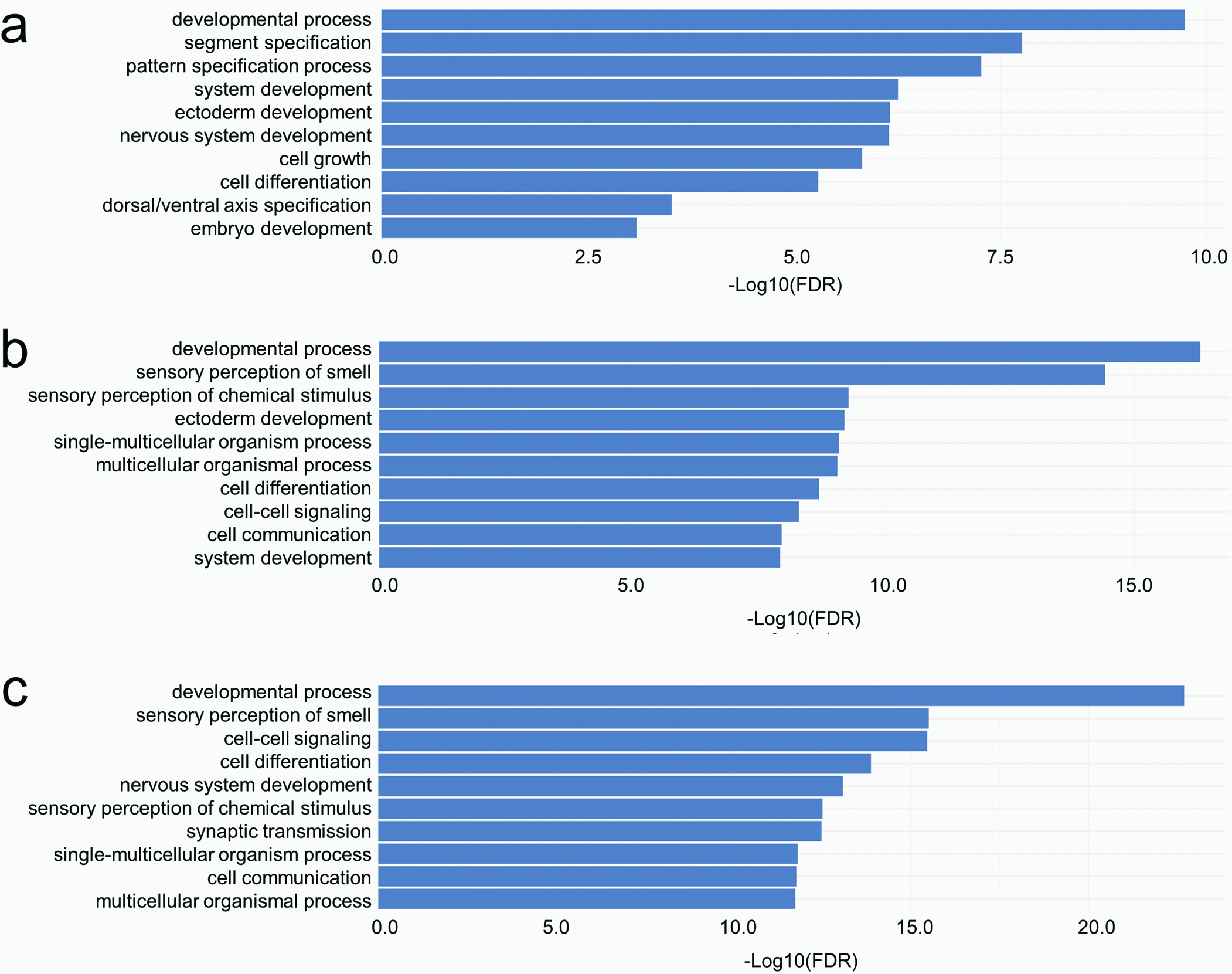

Supplement: Supplementary file 3 — Supplementary Fig 3 [file 41420_2019_184_MOESM3_ESM.tif]
