# Supplementary material for: A six-inhibitor culture medium for improving naïve-type pluripotency of porcine pluripotent stem cells
Source: Cell Death Discov. 2019 Jun 17;5:104. doi: 10.1038/s41420-019-0184-4 (PMC6579764; doi:10.1038/s41420-019-0184-4)
Supplement: Supplementary file 5 — Supplemental Material File #1 [file 41420_2019_184_MOESM5_ESM.docx]

**Supplementary Figure Legends**

**Title Page**

**A six-inhibitor culture medium for improving naïve-type pluripotency of porcine pluripotent stem cells**

Running title

*Improved conditions for porcine pluripotency*

Ye Yuan^1, 2, 3^*, Jinkyu Park^1,^ ^2, 4^*, Yuchen Tian^1^, Jungmin Choi^5^, Rolando Pasquariello^3, 6^, Andrei P. Alexenko^1, 2^, Aihua Dai^1^, Susanta K. Behura^2^, R. Michael Roberts^1, 2^, Toshihiko Ezashi^1, 2^

^1^Bond Life Sciences Center, University of Missouri, Columbia, MO 65211 ^2^Division of Animal Sciences, University of Missouri, Columbia, MO 65211 ^3^Colorado Center for Reproductive Medicine, Lone Tree, CO 80124 ^4^Department of Internal Medicine, Yale School of Medicine, New Haven, CT 06510 ^5^Laboratory of Human Genetics and Genomics, The Rockefeller University, New York, NY 10065 ^6^Department of Agricultural and Environmental Sciences - Production, Landscape, Agroenergy, University of Milan, Milano 20133, Italy.

*Authors contributed equally to the work.

Correspondence: Toshihiko Ezashi, 240a Bond Life Sciences Center, University of Missouri, Columbia, MO 65211, tel: (573) 884-9601, fax: (573) 884-9676, [ezashit@missouri.edu](mailto:ezashit@missouri.edu)

**Supplementary Fig 1.** Live cell images of pESCLC at initial stages (**a**-**c**) and later stage in the respective conditions (**d**-**f**). Primary colonies were derived in F (**a**), FLB2i (**b**), and FL6i (**c**) conditions. Primary colonies under F condition were switched to FL6i conditions (**d**). The proliferative cells were maintained in FL6i condition continuously for 82 days (**f**) or transferred into FLB2i condition for 74 days (**e**). Bars, 500 µm (a, b, d-f) or 200 µm (c).

**Supplementary Fig 2.** Hierarchical clustering of gene expression profiles of three piPSC (Lv-piPSC, Epi-piPSC-FLB2i and Epi-piPSC-FL6i) and three pESCLC (pESCLC-F, pESCLC-FLB2i, pESCLC-FL6i) lines and primary blastocyst outgrowth (pESCLC-primary) samples in duplicate.

**Supplementary Fig 3.** Top 10 categories enriched in the Gene Ontology (GO) analysis of differentially expressed genes (DEG) among three pESCLC (pESCLC-F, pESCLC-FLB2i, pESCLC-FL6i) lines and primary blastocyst outgrowth (pESCLC-primary) samples. (**a**) Top 10 enriched GO categories by comparing pESCLC-Primary and pESCLC-F. (**b**) Top 10 enriched GO categories by comparing pESCLC-Primary vs pESCLC-FLB2i. (**c**) Top 10 enriched GO categories by comparing pESCLC-Primary vs pESCLC-FL6i.

**Supplementary Table 1**. (**a**) Medium composition of testing six conditions (1 to 6 in top row) to identify component that causes the differentiation of piPSC in NHSM. The ‘o’ indicates the component listed in the left column is included and ‘X’ indicates the component is omitted in each condition. (**b**) Definition of the acronyms and the composition of the media used in this study. (**c**) Definition of the cell line acronyms presented in this study.

**Supplementary Table 2.** Efficiency of the outgrowths derived from porcine embryos. **a**: number of outgrowths, **b**: number of embryos used, **c**: efficiency of the outgrowths

**Supplementary Table 3.** Summary of teratoma studies by the cell types examined. The studies of human iPSC (Epi-hiPSC) and lentiviral piPSC (Lv-piPSC-F) are also described elsewhere as shown the references. Two and three sublines of Epi-piPSC-FLB2i and Epi-piPSC-FL6i, respectively were transplanted to immunodeficient mice (n; mice number) and solid tumors were collected after the days indicated.

**Supplementary Table 4.** Primer sequence information used in RT-PCR and qPCR experiments.
