# Supplementary material for: A six-inhibitor culture medium for improving naïve-type pluripotency of porcine pluripotent stem cells
Source: Cell Death Discov. 2019 Jun 17;5:104. doi: 10.1038/s41420-019-0184-4 (PMC6579764; doi:10.1038/s41420-019-0184-4)
Supplement: Supplementary file 6 — Supplemental Material File #2 [file 41420_2019_184_MOESM6_ESM.docx]

**Supplementary Tables**

**Title Page**

**A six-inhibitor culture medium for improving naïve-type pluripotency of porcine pluripotent stem cells**

Running title

*Improved conditions for porcine pluripotency*

Ye Yuan^1, 2, 3^*, Jinkyu Park^1,^ ^2, 4^*, Yuchen Tian^1^, Jungmin Choi^5^, Rolando Pasquariello^3, 6^, Andrei P. Alexenko^1, 2^, Aihua Dai^1^, Susanta K. Behura^2^, R. Michael Roberts^1, 2^, Toshihiko Ezashi^1, 2^

^1^Bond Life Sciences Center, University of Missouri, Columbia, MO 65211 ^2^Division of Animal Sciences, University of Missouri, Columbia, MO 65211 ^3^Colorado Center for Reproductive Medicine, Lone Tree, CO 80124 ^4^Department of Internal Medicine, Yale School of Medicine, New Haven, CT 06510 ^5^Laboratory of Human Genetics and Genomics, The Rockefeller University, New York, NY 10065 ^6^Department of Agricultural and Environmental Sciences - Production, Landscape, Agroenergy, University of Milan, Milano 20133, Italy.

*Authors contributed equally to the work.

Correspondence: Toshihiko Ezashi, 240a Bond Life Sciences Center, University of Missouri, Columbia, MO 65211, tel: (573) 884-9601, fax: (573) 884-9676, [ezashit@missouri.edu](mailto:ezashit@missouri.edu)

**a**

| **Testing conditions** | **1. Base** | **2. 2i (-)** | **3. TGFB1 (-)** | **4. p38i (-)** | **5 JNKi (-)** | **6 BMPi**  **(-)** |
| --- | --- | --- | --- | --- | --- | --- |
| IGF1 | o | o | o | o | o | o |
| ROCKi | o | o | o | o | o | o |
| FGF2 | o | o | o | o | o | o |
| LIF | o | o | o | o | o | o |
| GSK3i | X | X | o | o | o | o |
| MAP2K1i | X | X | o | o | o | o |
| TGFB1 | X | o | X | o | o | o |
| p38i | X | o | o | X | o | o |
| JNKi | X | o | o | o | X | o |
| BMPi | X | o | o | o | o | X |

**b**

| **Medium Abbreviation** | **Description** |
| --- | --- |
| **NHSM** | naïve human stem cell medium [^27^](#_ENREF_27) |
| **F** | primed ESC medium supplemented with FGF2 [^7^](#_ENREF_7)^,^[^34^](#_ENREF_34) |
| **FLB2i** | a condition to generate transgene-free intermediate pluripotent state piPSC consisting of LIF, FGF2, BMP4, GSK3A inhibitor (GSK3i, CHIR99021), TGFB1 inhibitor (TGFBi, A83-01) [^19^](#_ENREF_19) in NHSM based medium. |
| **FL6i** | a modified NHSM medium consisting of FGF2, LIF, MAPK14 inhibitor (p38i, BIRB796), MAPK8 inhibitor (JNKi, SP600125), TGFB1 inhibitor, GSK3A inhibitor, MAP2K1 inhibitor (MEKi PD0325901), BMP inhibitor (BMPi, LDN193189). |

| **Medium**  **Reprogram Method/ Cell Source** | **F** | **FLB2i** | **FL6i** |
| --- | --- | --- | --- |
| **Lentiviral Vector** | Lv-piPSC-F |  | Lv-piPSC-FL6i |
| **Episomal Plasmid** | Epi-piPSC-F | Epi-piPSC-FLB2i | Epi-piPSC-FL6i |
| **Blastocyst** | pESCLC-primary / pESCLC-F | pESCLC-FLB2i | pESCLC-FL6i |

**c**

**Supplementary Table 1**. (**a**) Medium composition of testing six conditions (1 to 6 in top row) to identify component that causes the differentiation of piPSC in NHSM. The ‘o’ indicates the component listed in the left column is included and ‘X’ indicates the component is omitted in each condition. (**b**) Definition of the acronyms and the composition of the media used in this study. (**c**) Definition of the cell line acronyms presented in this study.

| **Seeding methods** | | **Embryos** | |
| --- | --- | --- | --- |
|  |  | **Non-hatched embryos**  **a/b (%)^c^** | **Hatched embryos**  **a/b (%)^c^** |
| **Whole**  **embryos** | **F** | 2/86 (2.3%) | 9/35 (25.7%) |
|  | **FL6i** | 1/113 (0.9%) | 6/59 (10.2%) |
| **Immunosurgery** | **F** | 3/129 (2.3%) | 10/63 (15.9%) |
|  | **FL6i** | 0/117 (0%) | 1/29 (3.4%) |

**Supplementary Table 2.** Efficiency of the outgrowths derived from porcine embryos. **a**: number of outgrowths, **b**: number of embryos used, **c**: efficiency of the outgrowths

| **Cell type** | **references or origin** | **transplanted cell lines** | **days of teratoma collections** |
| --- | --- | --- | --- |
| Epi-hiPSC | [^71^](#_ENREF_71)^,^[^72^](#_ENREF_72) | human iPSC, MRuc8i | 46 (n = 2) |
| Epi-piPSC-FLB2i | this study | porcine iPSC, B5 | 38 (n = 2) |
| Epi-piPSC-FLB2i | this study | porcine iPSC, B7 | 40 (n =2) |
| Epi-piPSC-FL6i | this study | porcine iPSC, C10 | 62 (n = 1) |
| Epi-piPSC-FL6i | this study | porcine iPSC, C12 | 62 (n = 1) |
| Epi-piPSC-FL6i | this study | porcine iPSC, C13 | 62 ( n = 1) |
| Lv-piPSC-F | [^34^](#_ENREF_34) | porcine iPSC, ID6 | 90 (n = 2) |
| Lv-piPSC-FL6i | [^34^](#_ENREF_34), this study | porcine iPSC, ID6-FL6i | 39 (n = 2) |

**Supplementary Table 3.** Summary of teratoma studies by the cell types examined. The studies of human iPSC (Epi-hiPSC) and lentiviral piPSC (Lv-piPSC-F) are also described elsewhere as shown the references. Two and three sublines of Epi-piPSC-FLB2i and Epi-piPSC-FL6i, respectively were transplanted to immunodeficient mice (n; mice number) and solid tumors were collected after the days indicated.

| **Gene** | **Primer Sequence** | **Reference** |
| --- | --- | --- |
| *hGAPDH* | ACCACAGTCCATGCCATCAC | [1] |
|  | TCCACCACCCTGTTGCTGTA |  |
| *hPOU5F1* | CCCCAGGGCCCCATTTTGGTACC | [1] |
|  | ACCTCAGTTTGAATGCATGGGAGAGC |  |
| *hSOX2* | TTCACATGTCCCAGCACTACCAGA | [1] |
|  | TCACATGTGTGAGAGGGGCAGTGTGC |  |
| *hKLF4* | ACCCATCCTTCCTGCCCGATCAGA | [1] |
|  | TTGGTAATGGAGCGGCGGGACTTG |  |
| *plasmid-POU5F1* | CATTCAAACTGAGGTAAGGG | [1] |
|  | TAGCGTAAAAGGAGCAACATAG |  |
| *plasmid-SOX2* | TTCACATGTCCCAGCACTACCAGA | [1] |
|  | TTTGTTTGACAGGAGCGACAAT |  |
| *plasmid-LIN28* | AGCCATATGGTAGCCTCATGTCCGC | [1] |
|  | TAGCGTAAAAGGAGCAACATAG |  |
| *pGAPDH* | CTCAACGGGAAGCTCACTGG | [2] |
|  | CATTGTCGTACGAGGAAATGAGC |  |
| *pPOU5F1* | GCTGACAACAACGAGAATCTGC | [2] |
|  | ACGCGGACCACATCCTTCTCTAG |  |
| *pSOX2* | AATGCGCACAGCGCGGCT | [2] |
|  | GCCCATGGA ACCGAGCGT |  |
| *pNANOG* | CCGAAGCATCCATTTCCAGCG | [2] |
|  | GGTATTCTGTACTGGCTGAGCC |  |
| *pMYC* | GCCAAAAGGTCGGAATCGGGG | [2] |
|  | CGCAGCACGTCTTTTTCTGACAC |  |
| *pKLF4* | CCATGGGCCAAACTACCCAC | [2] |
|  | TGGGGTCAACACCATTCCGT |  |
| *pCRABP2* | CTGACCATGACGGCAGATGA | [2] |
|  | CCCCAGAAGTGACCGAAGTG |  |
| *pDESMIN* | CCTCAACTTCCGAGAAACAAGC | [2] |
|  | TCACTGACGACCTCCCCATC |  |
| *pNESTIN* | CAGCAAAGAACTGGAGTTCATCACTCT | [2] |
|  | AGGAAAAGCTGGGGTCCTCTTCAG |  |
| *pACTC1* | ATT TGC GGT GGA CGA TGG A | [3] |
|  | CAG GTA TTG CTG ATC GCA TGC A |  |
| *pDPPA3* | CTGAGTAGGTTGAGCCCACA | [3] |
|  | CCAAAAGAGGCAAAACCTGA |  |
| *pZFP42* | TTTCTGAGTACGTGCCAGGCAA | [3] |
|  | GAACGGAGAGACGCTTTCTCAGAG |  |
| *pSOX17* | CAG ACG TCG GGG TAG TTA CAG | [3] |
|  | CGC ACG GAG TTT GAA CAA TA |  |
| *pGATA6* | TTC TCG GGA TTA GCG CTC TC | [3] |
|  | CAG GAA ACG AAA ACC TAA GAG CAT |  |
| *pPAX6* | CAG CTT CAC CAT GGC AA ATA | [3] |
|  | GGG AAA TGA GTC CTG TGG AA |  |
| *pAFP* | ACT TCT TGC TCT TGG CCT TGG | [4] |
|  | CGC GTT TCT GGT TGC TTA CAC |  |

**Supplementary Table 4. Primer sequence information used in RT-PCR and qPCR experiments.**

**References of Supplementary Table 4**

1. Okita, K. *et al.* A more efficient method to generate integration-free human iPS cells. *Nat Methods* **8**, 409-412 (2011).

2. Ezashi, T. *et al.* Derivation of induced pluripotent stem cells from pig somatic cells. *Proceedings of the National Academy of Sciences of the United States of America* **106**, 10993-10998 (2009).

3. Rodríguez, A., Allegrucci, C. & Alberio, R. Modulation of pluripotency in the porcine embryo and iPS cells. *PloS one* **7**, e49079 (2012).

4. Park, J. K. *et al.* Primed pluripotent cell lines derived from various embryonic origins and somatic cells in pig. *PloS one* **8**, e52481 (2013).
